# Genome-wide Association Study of Change in Fasting Glucose over time in 13,807 non-diabetic European Ancestry Individuals

**DOI:** 10.1038/s41598-019-45823-7

**Published:** 2019-07-01

**Authors:** Ching-Ti Liu, Jordi Merino, Denis Rybin, Daniel DiCorpo, Kelly S. Benke, Jennifer L. Bragg-Gresham, Mickaël Canouil, Tanguy Corre, Harald Grallert, Aaron Isaacs, Zoltan Kutalik, Jari Lahti, Letizia Marullo, Carola Marzi, Laura J. Rasmussen-Torvik, Ghislain Rocheleau, Rico Rueedi, Chiara Scapoli, Niek Verweij, Nicole Vogelzangs, Sara M. Willems, Loïc Yengo, Stephan J. L. Bakker, John Beilby, Jennie Hui, Eero Kajantie, Martina Müller-Nurasyid, Wolfgang Rathmann, Beverley Balkau, Sven Bergmann, Johan G. Eriksson, Jose C. Florez, Philippe Froguel, Tamara Harris, Joseph Hung, Alan L. James, Maryam Kavousi, Iva Miljkovic, Arthur W. Musk, Lyle J. Palmer, Annette Peters, Ronan Roussel, Pim van der harst, Cornelia M. van Duijn, Peter Vollenweider, Inês Barroso, Inga Prokopenko, Josée Dupuis, James B. Meigs, Nabila Bouatia-Naji

**Affiliations:** 1 Department of Biostatistics, Boston University School of Public Health, Boston, Massachusetts, MA 02118 USA; 2 Center for Genomic Medicine, Massachusetts General Hospital, Boston, MA USA; 3 Programs in Metabolism and Medical & Population Genetics, Broad Institute of MIT and Harvard, Cambridge, MA USA; 4 Diabetes Unit, Massachusetts General Hospital, Boston, MA USA; 5 Bloomberg School of Public Health, Johns Hopkins University, Baltimore, MD USA; 6 Kidney Epidemiology and Cost Center, Department of Internal Medicine - Nephrology, University of Michigan, Ann Arbor, MI 48109 USA; 7 CNRS UMR 8199, European Genomic Institute for Diabetes (EGID), Institut Pasteur de Lille, University of Lille, 59000 Lille, France; 8 Institute of Social and Preventive Medicine, Lausanne University Hospital, Lausanne, Switzerland; 9 Department of Computational Biology, University of Lausanne, Lausanne, Switzerland; 10 Swiss Institute of Bioinformatics, Lausanne, Switzerland; 11 Research Unit of Molecular Epidemiology, Institute of Epidemiology, Helmholtz Zentrum München Research Center for Environmental Health, Neuherberg, Germany; 12 German Center for Diabetes Research (DZD), Neuherberg, Germany; 13 Genetic Epidemiology Unit, Department of Epidemiology, Erasmus University Medical Center, Rotterdam, The Netherlands; 14 CARIM School for Cardiovascular Diseases, Maastricht Centre for Systems Biology (MaCSBio) and Department of Biochemistry, Maastricht University, Maastricht, The Netherlands; 15 Helsinki Collegium for Advanced Studies, University of Helsinki, Helsinki, Finland; 16 Department of Psychology and Logopedics, University of Helsinki, Helsinki, Finland; 17 Department of Life Sciences and Biotechnology, University of Ferrara, Ferrara, Italy; 18 Department of Preventive Medicine, Northwestern University Feinberg School of Medicine, Chicago, IL USA; 19 Preventive and Genomic Cardiology, McGill University Health Centre and Research Institute, Montreal, Quebec Canada; 20 The Charles Bronfman Institute for Personalized Medicine, Icahn School of Medicine at Mount Sinai, New York, NY USA; 21 The Icahn Institute for Genomics and Multiscale Biology, Icahn School of Medicine at Mount Sinai, New York, NY USA; 22 Department of Genetics and Genomic Sciences, Icahn School of Medicine at Mount Sinai, New York, NY USA; 23 University Medical Center Groningen, Department of Cardiology, University of Groningen, Groningen, The Netherlands; 24 Maastricht University, Department of Epidemiology, Cardiovascular Research Institute Maastricht (CARIM) & Maastricht Centre for Systems Biology (MaCSBio), Maastricht, The Netherlands; 25 University of Groningen, University Medical Center Groningen, Department of Internal Medicine, Groningen, The Netherlands; 26 PathWest Laboratory Medicine of Western Australia, Nedlands WA, 6009 Australia; 27 School of Pathology and Laboratory Medicine, The University of Western Australia, Nedlands WA, 6009 Australia; 28 Busselton Population Medical Research Foundation, Sir Charles Gairdner Hospital, Nedlands WA, Australia; 29 School of Population and Global Health, The University of Western Australia, Nedlands WA, 6009 Australia; 30 National Institute for Health and Welfare, Helsinki, Finland; 31 Institute of Genetic Epidemiology, Helmholtz Zentrum München, German Research Center for Environmental Health, Neuherberg, Germany; 32 Department of Medicine I, University Hospital Grosshadern, Ludwig-Maximilians-Universität, Munich, Germany; 33 Institute of Medical Informatics, Biometry and Epidemiology, Chair of Genetic Epidemiology, Ludwig-Maximilians-Universität, Munich, Germany; 34 DZHK (German Centre for Cardiovascular Research), partner site Munich Heart Alliance, Munich, Germany; 35 Institute for Biometrics and Epidemiology, German Diabetes Centre, Leibniz Center for Diabetes Research at Heinrich Heine University Düsseldorf, Auf’m Hennekamp 65, 40225 Düsseldorf, Germany; 36 CESP Centre for Research in Epidemiology and Population Health, Villejuif, France; 37 Univ. Paris-Saclay, Univ. Paris Sud, UVSQ, UMRS 1018, F-94807 Villejuif, France; 38 INSERM U1018, CESP, Renal and Cardiovascular Epidemiology, UVSQ-UPS, Villejuif, France; 39 Department of Integrative Biomedical Sciences, University of Cape Town, Cape Town, South Africa; 40 Department of General Practice and Primary Health Care, University of Helsinki, Helsinki, Finland; 41 Folkhälsan Research Centre, Helsinki, Finland; 42 Department of Medicine, Harvard Medical School, Boston, MA 02115 USA; 43 Department of Genomics of Common Disease, Imperial College London, London, United Kingdom; 44 National Institute on Aging, Laboratory of Epidemiology and Population Sciences in Intramural Research Program, Baltimore, MD USA; 45 School of Medicine and Pharmacology, The University of Western Australia, Nedlands WA, 6009 Australia; 46 Department of Pulmonary Physiology, Sir Charles Gairdner Hospital, Nedlands WA, 6009 Australia; 47 Department of Epidemiology, Erasmus MC, University Medical Center Rotterdam, Rotterdam, The Netherlands; 48 Department of Epidemiology, University of Pittsburgh, Pittsburgh, PA USA; 49 School of Public Health, University of Adelaide, Adelaide, Australia; 50 Institute of Epidemiology, Helmholtz Zentrum München Research Center for Environmental Health, Neuherberg, Germany; 51 INSERM U1138 (équipe 2: Pathophysiology and Therapeutics of Vascular and Renal Diseases Related to Diabetes, Centre de Recherches des Cordeliers), Paris, France; 52 Univ. Paris 7 Denis Diderot, Sorbonne Paris Cité, France; 53 AP-HP, DHU FIRE, Department of Endocrinology, Diabetology, Nutrition, and Metabolic Diseases, Bichat Claude Bernard Hospital, Paris, France; 54 University of Groningen, University Medical Center Groningen, Department of Genetics, Groningen, The Netherlands; 55 Durrer Center for Cardiogenetic Research, ICIN-Netherlands Heart Institute, Utrecht, The Netherlands; 56 Department of Medicine, Internal Medicine, Lausanne University Hospital (CHUV), Lausanne, Switzerland; 57 Wellcome Sanger Institute, Wellcome Genome Campus, Cambridge, UK; 58 Department of Medicine, Imperial College London, London, United Kingdom; 59 Wellcome Centre for Human genetics, University of Oxford, Oxford, United Kingdom; 60 Oxford Centre for Diabetes, Endocrinology and Metabolism, University of Oxford, Oxford, United Kingdom; 61 The National Heart, Lung, and Blood Institute’s Framingham Heart Study, Framingham, MA USA; 62 Division of General Internal Medicine, Massachusetts General Hospital, Boston, MA USA; 63 INSERM, UMR970 Paris Cardiovascular Research Center (PARCC), Paris, F-75015 France; 64 Paris-Descartes University, Sorbonne Paris Cité, Paris, 75006 France

**Keywords:** Medical genetics, Type 2 diabetes

## Abstract

Type 2 diabetes (T2D) affects the health of millions of people worldwide. The identification of genetic determinants associated with changes in glycemia over time might illuminate biological features that precede the development of T2D. Here we conducted a genome-wide association study of longitudinal fasting glucose changes in up to 13,807 non-diabetic individuals of European descent from nine cohorts. Fasting glucose change over time was defined as the slope of the line defined by multiple fasting glucose measurements obtained over up to 14 years of observation. We tested for associations of genetic variants with inverse-normal transformed fasting glucose change over time adjusting for age at baseline, sex, and principal components of genetic variation. We found no genome-wide significant association (P < 5 × 10^−8^) with fasting glucose change over time. Seven loci previously associated with T2D, fasting glucose or HbA1c were nominally (P < 0.05) associated with fasting glucose change over time. Limited power influences unambiguous interpretation, but these data suggest that genetic effects on fasting glucose change over time are likely to be small. A public version of the data provides a genomic resource to combine with future studies to evaluate shared genetic links with T2D and other metabolic risk traits.

## Introduction

Type 2 diabetes mellitus (T2D), a disease characterized by persistent hyperglycemia, is a common and heritable complex disease affecting the health of millions of people worldwide^1^. Estimates from the World Health Organization indicate that 8.5% of the adult population had T2D in 2016, and this prevalence has been steadily increasing during the last three decades^2^.

Prospective epidemiological studies have demonstrated that the risk of T2D starts even in the normal fasting glucose range and exponentially increases in pre-diabetic ranges^3–7^. Relevant physiological perturbations producing a slow utilization of fasting glucose are likely to be present at stages of the disease as early as a decade before diagnosis^8^. The etiological causes of early glycemic perturbations are likely to be triggered by environmental and lifestyle factors^9,10^, but the precise biological mechanisms underpinning why people differently progress to hyperglycemia are unknown.

Recent large-scale genetic association meta-analyses have uncovered genetic variants cross-sectionally associated with T2D and related glycemic traits^11–16^. However, prospective data for genetic variant association discovery are scarce and findings have been inconsistent^17–19^. In this study, we conducted the largest genome-wide association study (GWAS) to date to identify genetic variants associated with fasting glucose changes over up to 14 years in 13,807 non-diabetic participants of European descent from nine cohorts.

## Results

We included a total of 13,807 participants of European descent and free of diabetes at baseline and during the entire follow-up period with repeated fasting glucose levels measured at least at two time points over up to 14 years from nine cohorts. Characteristics of the study sample and follow-up, phenotype, and genotype information are presented in Table S1. The participants’ average age at baseline for each cohort ranged from 41 to 70 years old. The follow-up time varied by cohort, on average ranging from 5 to 25 years. The slope of fasting glucose was calculated based on available fasting glucose measurements during the follow-up and then the slopes were inverse normal transformed within each cohort, for harmonization. We refer to this transformed slope as fasting glucose change over time. At baseline, cohort-specific average fasting glucose ranged from 4.9 to 6.2 mmol/L in men and 4.8 to 5.9 mmol/L in women, respectively. Our primary analysis included all available participants. In an exploratory analysis, we investigated whether stratifying our sample by cohorts with long-term follow-up [≥10 years] or short-term follow-up [<10 years] identified pertinent signals.

**Figure 1:**
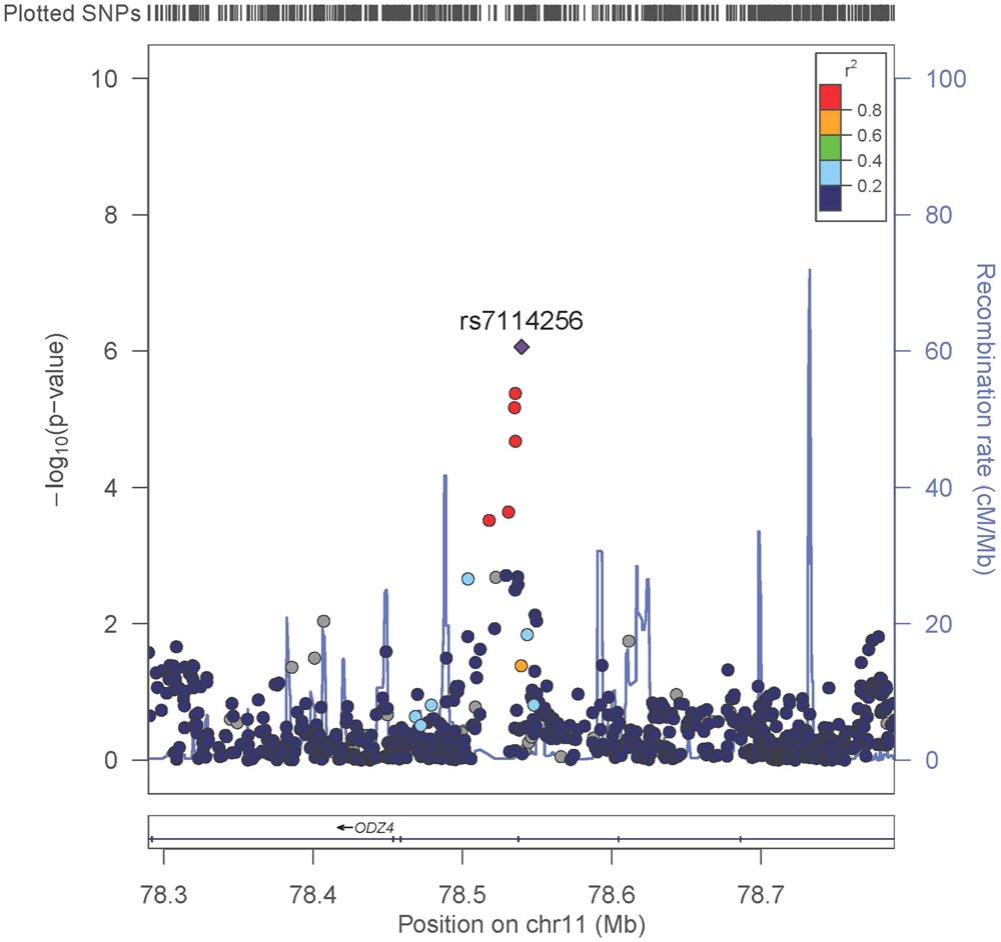
Regional association plot of rs7114256. Results from 500 kb regional associations for fasting glucose change over time, centered at rs7114256. The x axis denotes genomic position build 36 and the y axis denotes the −log(P-value) and recombination rate (blue line). The purple diamond symbol represents the most-associated SNP within the region, rs7114256. The color of each symbol indicates the LD value with rs7114256 based on the HapMap2 CEU sample.

In a genome-wide association meta-analysis we did not find evidence of genetic variants associated with fasting glucose change over time at genome-wide significance level (*P* < 5 × 10^−8^) (Supplemental Figs 1–3) nor evidence of inflated signals (Supplemental Figs 4–6) in the primary analysis including the entire sample or the sensitivity analysis. For the analysis with all samples, the most significant association with fasting glucose changes over time was an intronic variant at the *ODZ4* locus (rs7114256; *P* = 8.78 × 10^−7^, Table 1, Fig. 1). There were five other suggestively associated (*P* < 5 × 10^−6^) variants for fasting glucose changes over time in three loci whose closest reference genes including *ALLC* (rs606243), *NUDT12* (rs17496593, rs17496653, rs17562893) and *ODZ4* (rs7103693) (Table 1, Supplemental Figs 7–8). In our exploratory analysis stratifying cohorts by follow-up time, there were a few suggestively associated variants with fasting glucose changes over time (Supplemental Tables 5–6). These included four loci whose closest reference genes were *SNX16*, *BEGFA*, *GATA3* and *CDKAL1* from short follow-up analysis with sample size up to 8,195 and ten loci whose closest reference genes were *HCRTR2*, *WRN*, *SEPT9*, *SLC35B3*, *FAM84A*, *GRM8*, *MPP6*, *BAMBI*, *SSB*, and *C8orf31* from long follow-up analysis with sample size up to 3,669.

**Table 1:** Genome-wide association results for genetic variants with an association p-value < 5 × 10^−6^.

SNP	Chr	BP^a^	EA^b^	NEA^b^	EAF^b^	Beta	SE	P	Direction^c^	HetPVal^d^	N	RefGen^e^
rs7114256	11	78539553	A	G	0.92	0.129	0.03	8.78E-07	+++++−?++	0.74	13,003	*ODZ4*
rs606243	2	4487817	A	G	0.74	−0.078	0.02	1.42E-06	------??-	0.36	11,862	*ALLC*
rs17496593	5	104254353	A	C	0.91	−0.111	0.02	2.12E-06	-----+?--	0.21	13,005	*NUDT12*
rs17496653	5	104255187	A	G	0.09	0.110	0.02	2.58E-06	+++++−?++	0.20	13,005	*NUDT12*
rs17562893	5	104266799	T	G	0.09	0.108	0.02	3.78E-06	+++++−?++	0.19	12,994	*NUDT12*
rs7103693	11	78535307	T	C	0.08	−0.120	0.03	4.19E-06	-----+?--	0.79	13,003	*ODZ4*

^a^Physical Position (base pair) in build 36.

^b^EA: effect allele, NEA: non-effect allele, EAF: effect allele frequency.

^c^The sign of EA effect and the order of Cohorts are BHS, COLAUS, DESIR, ERGO, FHS, HBCS, KORA, PREVEND, SARDIANA.

^d^HetPVal: P-value for testing for heterogeneity.

^e^RefGen: closest reference gene.

Next, we investigated whether genetic variants available in our GWAS previously associated with T2D prevalence (82 SNPs)^15^ and cross-sectional glycemic traits based on our primary analysis with all available sample, including fasting glucose (32 SNPs)^13^, and HbA1c (58 SNPs)^16^, associated with fasting glucose change over time^13,15,16^. For T2D associated genetic variants^15^, we showed evidence of a nominal significant association between a variant at *CDKN2A/B* loci (rs10965248, *P* = 0.0192) and longitudinal fasting glucose change. In addition, two loci previously associated with cross-sectional FG in the latest GWAS^13^ for fasting glucose associated with longitudinal fasting glucose changes (*GRB10*; rs6943153, *P* = 0.0019 and *PDX1*; rs11619319, *P* = 0.0114), as well as five loci previously associated with HbA1c in the latest GWAS for HbA1c^16^ including *TMC6 (*rs2073285, *P* = 0.0019), *CDH3* (rs4783565, *P* = 0.0057), *ABO (*rs579459, *P* = 0.0082), *PDX1 (*rs11619319, *P* = 0.0114), and *HK1 (*rs10823343, *P* = 0.0390) (Table 2, Supplemental Tables 2–4). After Bonferroni correction for conduct of 82, 32, and 58 tests for T2D risk, fasting glucose, and HbA1c, respectively none of these signals remained significant.

**Table 2:** Association results of the genetic variants showing a nominal significant signal for fasting glucose change (p < 0.05) in known T2D or glycemic trait loci^a^.

SNP	Chr	BP^b^	Locus	EA^b^	NEA^b^	EAF^b^	Beta	SE	P	Direction^c^	HetPVal^d^	N	HetISq
**Fasting glucose loci**													
rs6943153	7	50759073	*GRB10*	T	C	0.30	−0.044	0.01	0.002	-------+-	0.4257	13,800	1
rs11619319	13	27385599	*PDX1*	A	G	0.77	0.039	0.02	0.011	+++++−+++	0.6785	13,807	0
**Type 2 diabetes loci**													
rs10965248^e^	9	22122878	*CDKN2A/B*	A	G	0.18	−0.040	0.02	0.019	?--+--+--	0.06878	12,523	47
**HbA1c loci**													
rs2073285	17	73628956	*TMC6*	T	C	0.20	0.079	0.03	0.002	+?++?+?+?	0.05396	5,098	57
rs4783565	16	67307691	*CDH3*	A	G	0.32	0.043	0.02	0.006	?++++++?+	0.4491	11,356	0
rs579459	9	135143989	*ABO*	T	C	0.78	−0.041	0.02	0.008	------+-+	0.2827	13,779	18
rs11619319	13	27385599	*PDX1*	A	G	0.77	0.039	0.02	0.011	+++++−+++	0.6785	13,807	0
rs10823343	10	70761019	*HK1*	A	G	0.75	−0.037	0.02	0.039	-+-----??	0.3766	8,809	6.7

^a^Scott *et al*.^13^, Scott *et al*.^15^, Wheeler *et al*.^16^.

^b^BP:Physical Position (base pair) in build 36. EA: effect allele, NEA: non-effect allele, EAF: effect allele frequency.

^c^The sign of EA effect and the order of Cohorts are BHS, COLAUS, DESIR, ERGO, FHS, HBCS, KORA, PREVEND, SARDIANA.

^d^HetPVal: P-value for testing for heterogeneity.

^e^Using proxy SNP rs10965250 (r2 = 0.97 with rs10965248).

**Figure 2:**
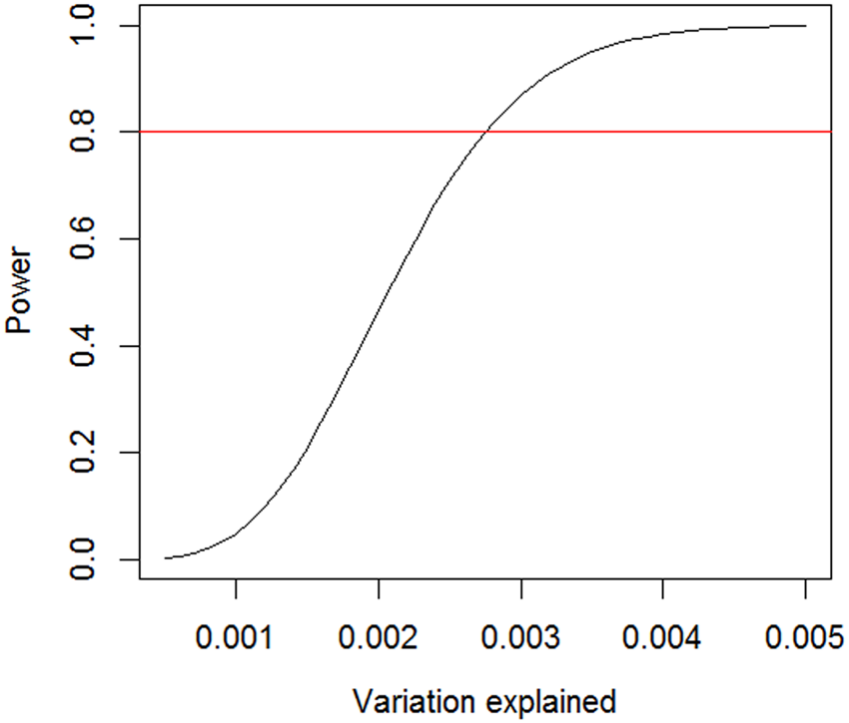
Power analysis. The relationship between power and variation explained in the trait of interest by a genetic variant with a sample size of 13,807 at a significance level of 5 × 10^−8^. The y-axis represents the power and the x-axis the variance explained by a genetic variant. The horizontal red line represents the power of 80%. This Figure shows that we had 80% power to detect a genetic variant that explained at least 0.28% of variation in fasting glucose change over time.

We conducted a power analysis with a sample size of 13,807 at the genome-wide significant threshold (5 × 10^−8^) to detect a genetic variant explaining at least 0.05% to 0.5% of the variation in the fasting glucose change over time. The results showed that our study has 80% power to detect genetic variants, which explain at least 0.28% of variation in change of fasting glucose over time (Fig. 2). This is equivalent to detect the genetic variants with minor allele frequency of 0.05 or 0.25 whose minimum effect corresponds to 0.17 or 0.09 standard deviation unit difference in the change of fasting glucose over time, respectively.

## Discussion

We tested whether common genetic variants were associated with fasting glucose change over time in a GWAS including 13,807 initially non-diabetic participants from nine cohorts of European descent with repeated fasting glucose measures over up to 14 years, We found three suggestive associated variants at sub genome significance level (near *ODZ4*, *ALLC*, and *NUDT12)*, and eight nominally associated previously known T2D-glycemia GWAS loci (*CDKN2A/B*, *GRB10*, *PDX1*, *TMC6*, *CDH3*, *ABO*, *PDX1*, and *HK1)* but none reached genome-wide significance for association or survived adjustment for multiple testing. We have placed the GWAS results data sets from this analysis on line at the Meta-Analyses of Glucose and Insulin-related traits Consortium (MAGIC) website (https://www.magicinvestigators.org) and T2D knowledge portal (http://www.type2diabetesgenetics.org) to provide a genomic resource to further combine with futures studies and evaluate shared genetic links with T2D and other metabolic risk traits.

To date, more than 120 genetic loci have been identified to be associated with cross-sectional glycemic outcomes in successive waves of large-scale genetic association studies^12,14,15^. These risk alleles are associated with glycemic phenotypes and predict incident T2D when aggregated into a genetic risk score^12,20,21^. However, findings from our study do not support that single common risk alleles have a substantive impact on longitudinal fasting glucose changes. If there are effects on glucose changes, they are likely to be small. This observation is in agreement with evidence from other intermediate phenotypes such as genetic variants associated with deterioration of lipid levels^22–25^, lung function^26,27^ or change in BMI^28–31^, where modest effects have been attributed to genetic factors on longitudinal trait changes, relative to single cross sectional trait measures. Regarding glycemic trajectories, no single genetic variants associated with glucose deterioration over time were detected in previous studies of European descent indivuals^17^ or in Han individuals^19^, although Han Chinese carrying a higher number of T2D increasing-risk variants showed a greater increase in FG over time compared with those carrying a lower number of T2D increasing-risk variants. One small single-cohort study identified five genomic regions associated at genome-wide significance with longitudinal change in fasting glucose (*GCKR*, *G6PC2*, *GCK*, *SLC30A8*, *MTNR1B*), but the study included diabetic individuals taking hypoglycemic medications, which almost certainly introduced confounding into genotype – glucose change associations^18^.

Our results may highlight the importance of environmental determinants of glycemic deterioration. Nevertheless, genetic determinants of changes in glycemia may remain relevant for people with rapid transition from pre-diabetes to diabetes, which the design of our study was not able to capture. A potential future strategy to identify these loci, if they exist, would be to focus on pre-diabetic individuals who progress to T2D and adjust for the effects of all known variants affecting cross-sectional blood glucose inter-individual variability^32^. A limitation to our study is that the meta-analysis only involved European participants, so the results may not be generalizable to other ancestry groups. The power of our study was relatively limited even though this is the largest existing meta-analysis of fasting glucose change. In addition, we identified challenges posed by phenotypic heterogeneity, e.g. different follow-up duration or different numbers of longitudinal data points. Larger sample sizes from new cohorts will be key to help confirm or refute the current findings. An alternative approach, if data are available in the future, would be to study longitudinal glycemia in large and homogeneous populations, with more homogeneous phenotypes especially with more consistent number of follow-up visits and similar follow-up duration to gain more statistical power.

In summary, a large GWAS did not identify common genetic variation genome-wide significantly associated with fasting glucose change over time. Such genetic effects, if present, are likely small. The data have been deposited as a public genetic epidemiological resource to aid the hunt for genetic determinants of T2D and its relevant physiology.

## Methods and Materials

### Study sample

We recruited in total 13,807 individuals of European descent free from T2D at baseline and during the entire follow-up period with repeated fasting glucose measurements at two or more time points from nine cohorts representing three continents (America, Europe and Australia). The participating cohorts include the Bogalusa Heart Study (BHS), the CoLaus study (COLAUS), the Data from the Epidemiological Study on the Insulin Resistance Syndrome study (DESIR), the Erasmus Rotterdam Gezondheid Onderzoek study (ERGO), Framingham Heart Study (FHS), the Helsinki Birth Cohort Study (HBCS), Cooperative Health Research in the Region of Augsburg (KORA), Prevention of Renal and Vascular End-stage Disease study (PREVEND), and the National Institute on Aging (NIA) SardiNIA Study (SARDINIA). The ethnicity information for each individual was based on questionnaires or assessed using genetic data (principal component analysis). Ethnic outliers detected by principal component analysis for European ethnicity were excluded from further analysis. Diabetes was defined as a fasting glucose level >7 mmol/l, or use of glucose lowering medication. The study conformed to the Declaration of Helsinki guidelines. Institutional Review Board and/or oversight committees approved the study in each participating cohort and all participants provided written informed consent (See Supplemental Text).

### Genotyping, imputation and quality control

Genotyping was conducted as specified in the Table S1. Each study imputed their genotype to ~2.5 million Phase 2 HapMap CEU SNPs with imputation software, either IMPUTE or MACH^33,34^. We applied a quality control filter by removing SNPs with a minor allele frequency less than 1% and those with an imputation quality threshold proper_info < 0.4 for cohorts using IMPUTE and r^2^ > 0.3 for cohorts using MACH. We used imputed allelic dosage in our association analysis.

### Phenotype

To calculate longitudinal fasting glucose slopes we used repeated FG measurements available in the longitudinal cohort studies. To harmonize FG measures, FG measures obtained from whole blood were converted to plasma levels by using a coefficient of 1.13 (FG in mmol/l). We then modelled the association for each individual between FG and duration of time between baseline measure and each follow-up measure. The resulting beta coefficients (slopes) were then pooled and inverse normal transformed. The transformed slopes were used as the trait ‘fasting glucose change’ for the genetic association analysis.

### Association Analysis and Meta-analysis

For each participating cohort, we conducted genome-wide association analysis with transformed longitudinal fasting glucose changes adjusting for age at baseline, sex, and principal components of genetic variation to account for population stratification using linear regression with additive genetic effects for cohorts with unrelated samples. We performed mixed-effect model analysis with random effect to account for sample relatedness for cohorts with related samples. We then conducted inverse variance weighted meta-analysis of cohort-specific association results using METAL^35^. In an exploratory analysis, we stratified our analyses by study follow-up time and classified each cohort as having a long follow-up time (≥10 years) or a short follow-up time (<10 years). We applied genomic control correction to control type I error^36^. SNPs with a meta-analysis p-value ≤ 5 × 10^−8^ were considered to be genome-wide significant.

### Interrogation of Published Loci for Type 2 Diabetes Related Traits

We tested the hypothesis that longitudinal fasting glucose slopes would be associated with previously-identified GWAS variants for T2D (128 SNPs)^15^, fasting glucose (32 SNPs)^13^, and HbA1c (60 SNPs)^16^. If the previously reported most-associated SNP was unavailable in the present analysis, we used a proxy SNP (LD r^2^ > 0.8) if available. After proxy searches we evaluated 172 loci, including 82 for T2D, 58 for HbA1c and 32 for FG. The Bonferroni corrected p-value threshold for these look-ups was set at 0.0003 (0.05/172).

### Post-hoc power calculation

We conducted a post-hoc power analysis using Quanto software to investigate the power to detect 0.05% to 0.5% percent variation in phenotype explained by a genetic variant with a sample size of 13,807 at the genome-wide significant threshold (5 × 10^−8^).

## Supplementary information

Supplemental Appendix

Supplemental Tables

## Data Availability

We have publicly deposited the summary results statistics on line at the Meta-Analyses of Glucose and Insulin-related traits Consortium (MAGIC) website (https://www.magicinvestigators.org) and T2D knowledge portal (http://www.type2diabetesgenetics.org).
