# Supplementary material for: Genome-wide Association Study of Change in Fasting Glucose over time in 13,807 non-diabetic European Ancestry Individuals
Source: Sci Rep. 2019 Jul 1;9:9439. doi: 10.1038/s41598-019-45823-7 (PMC6602949; doi:10.1038/s41598-019-45823-7)
Supplement: Supplementary file 1 — Supplemental Appendix [file 41598_2019_45823_MOESM1_ESM.pdf]

Supplementary appendix

Supplement to: Genome-wide Association Study of Change in Fasting Glucose over time in 13,807 non-diabetic European Ancestry Individuals

Ching-Ti Liu[1*], Jordi Merino[2,3,4*], Denis Rybin[1], Daniel DiCorpo[1], Kelly S Benke[5], Jennifer L Bragg-Gresham[6], Mickaël Canouil[7], Tanguy Corre[8,9,10], Harald Grallert[11,12], Aaron Isaacs[13,14], Zoltan Kutalik[8,10], Jari Lahti[15,16], Letizia Marullo[17], Carola Marzi[11,12], Laura J Rasmussen-Torvik[18], Ghislain Rocheleau[7,19,20,21,22], Rico Rueedi[9,10], Chiara Scapoli[17], Niek Verweij[23], Nicole Vogelzangs[24], Sara M Willems[13], Loïc Yengo[7], Stephan J L Bakker[25], John Beilby[26,27,28], Jennie Hui[26,27,28,29], Eero Kajantie[30], Martina Müller-Nurasyid[31,32,33,34], Wolfgang Rathmann[35], Beverley Balkau[36,37,38], Sven Bergmann[9,10,39], Johan G Eriksson[30,40,41], Jose C Florez[2,3,4,42], Philippe Froguel[7,43], Tamara Harris[44], Joseph Hung[28,45], Alan L James[28,45,46], Maryam Kavousi[47], Iva Miljkovic[48],Arthur W Musk[28,29,45], Lyle J Palmer[49], Annette Peters[12,50], Ronan Roussel[51,52,53], Pim van der harst[23,54,55], Cornelia M van Duijn[13], Peter Vollenweider[56], Inês Barroso[57], Inga Prokopenko[58,59,60], Josée Dupuis[1,61], James B Meigs[3,42,62], Nabila Bouatia-Naji[63,64]

* Contributed equally as first authors

Address for Correspondence:

Ching-Ti Liu, PhD. Department of Biostatistics, Boston University School of Public Health, 715 Albany St, Boston, MA 02118, USA. ctliu@bu.edu.

Nabila Bouatia-Naji, PhD. INSERM, UMR970 Paris Cardiovascular Research Center (PARCC), Paris F-75015, FRANCE. nabila.bouatia-naji@inserm.fr.

Supplemental Figure 1. Manhattan Plot for genome-wide association with the fasting glucose change over time <u>for all samples.</u> The red and blue lines represent the threshold of genome-wide significance (p-value of 5×10^{-8}) and suggestive significance (p-value of 5×10^{-6}).

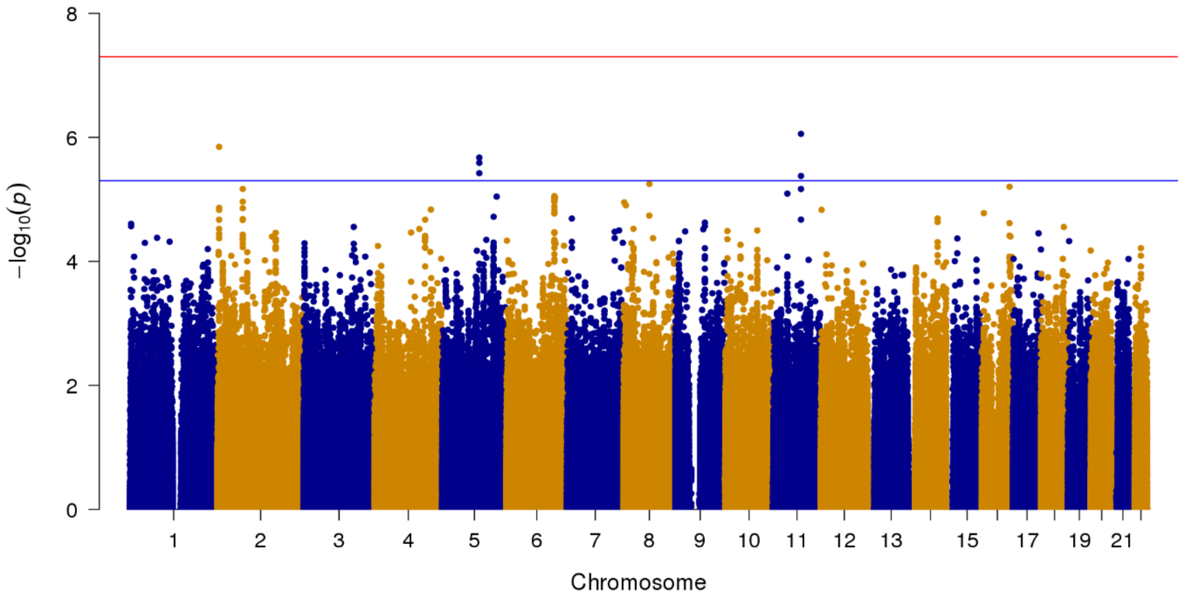

Supplemental Figure 2. Manhattan Plot for genome-wide association with the fasting glucose change over time for the cohorts with long-term follow-up time (i.e. at least 10 years). The red and blue lines represent the threshold of genome-wide significance (p-value of 5×10^{-8}) and suggestive significance (p-value of 5×10^{-6}).

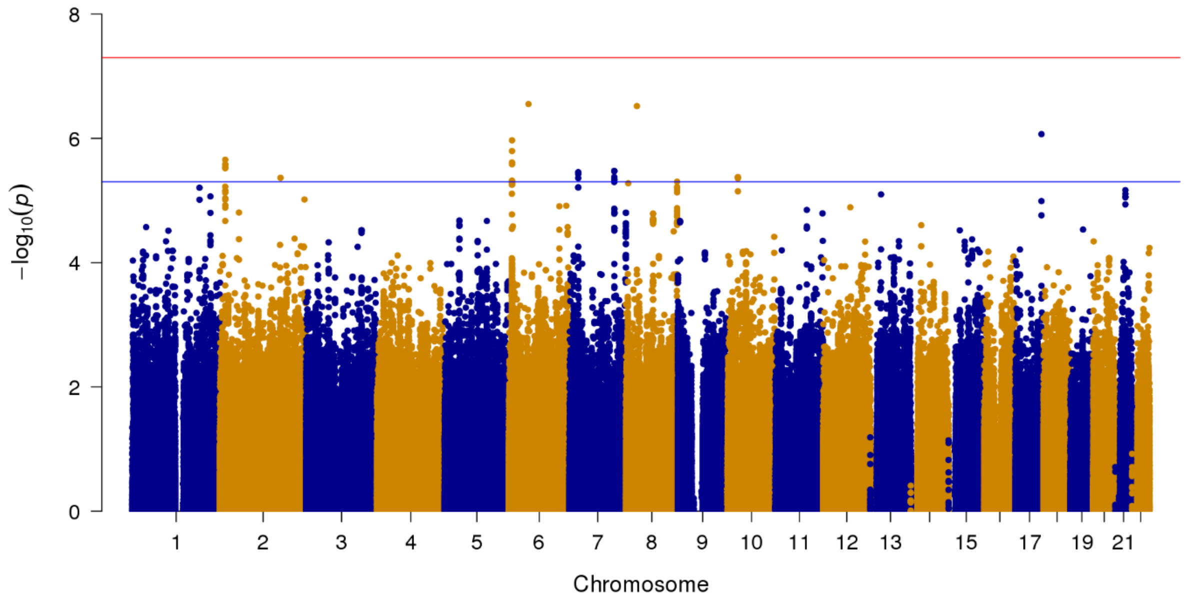

Supplemental Figure 3. Manhattan Plot for genome-wide association with the fasting glucose change over time for the cohorts with short-term follow-up time (i.e. less than 10 years). The red and blue lines represent the threshold of genome-wide significance (p-value of 5×10^{-8}) and suggestive significance (p-value of 5×10^{-6}).

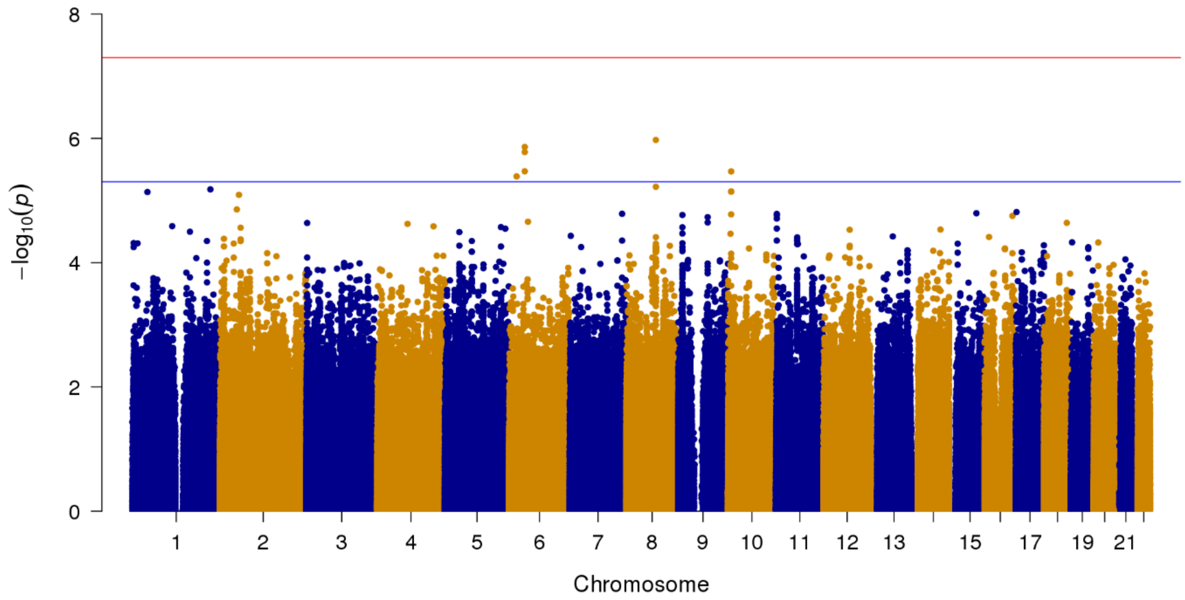

Supplemental Figure 4. Quantile-Quantile plot for genome-wide association with the fasting glucose change over time for all samples.

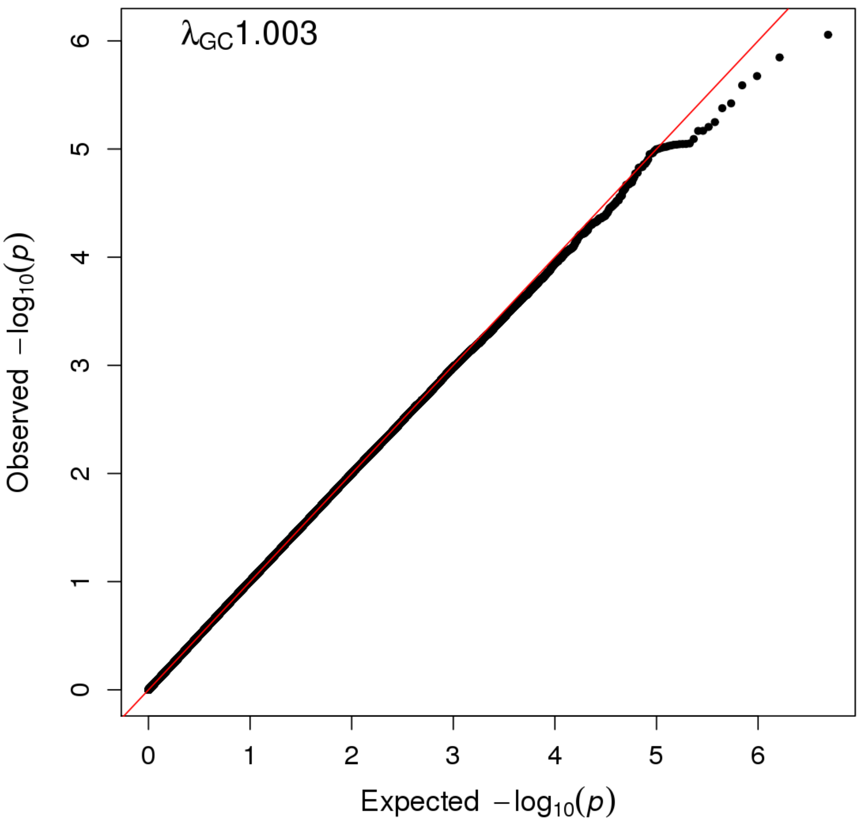

Supplemental Figure 5. Quantile-Quantile plot for genome-wide association with the fasting glucose change over time <u>for the cohorts with long-term follow-up time (i.e. at least 10 years).</u>

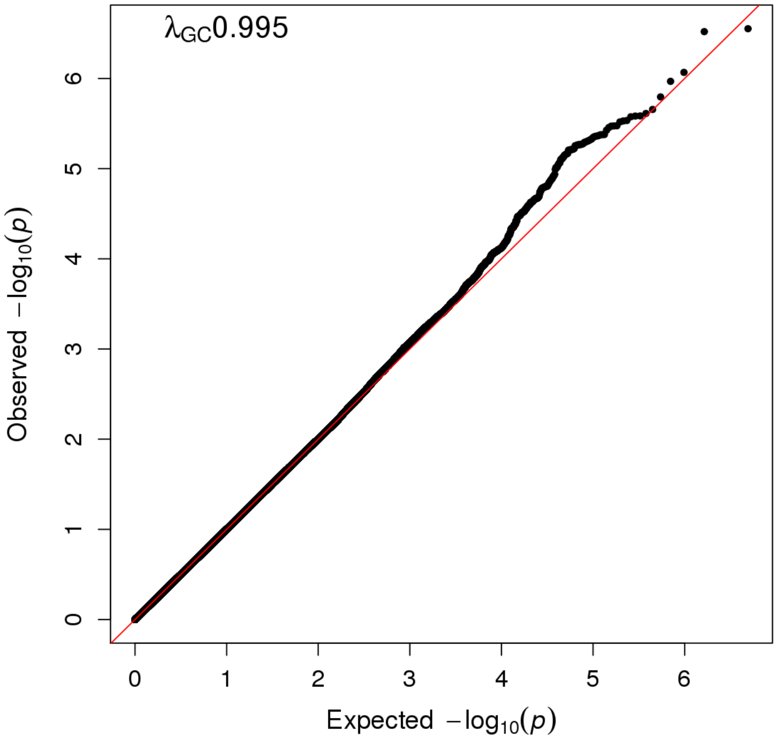

Supplemental Figure 6. Quantile-Quantile plot for genome-wide association with the fasting glucose change over time <u>for the cohorts with short-term follow-up time (i.e. less than 10 years).</u>

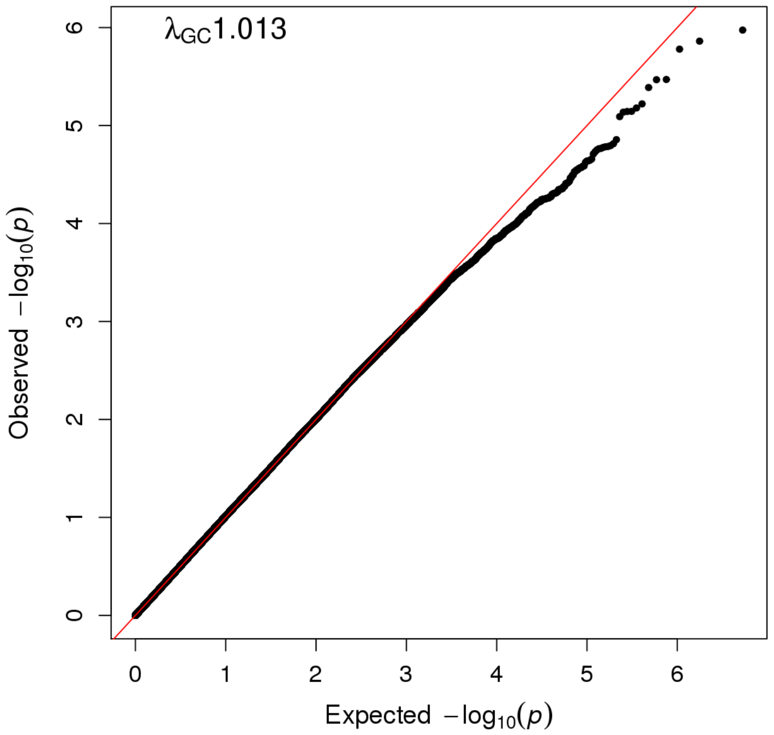

Supplemental Figure 7. Regional association plot of rs606243. Results from 500 kb regional association plot for fasting glucose change over time, centered at rs606243. The x axis denotes genomic position and the y axis denotes the –log(P-value) and recombination rate (blue line). The purple diamond symbol represents the most-associated SNP rs606243 within the region. The color of each symbol indicates its LD value with the top SNP based on the HapMap2 CEU sample.

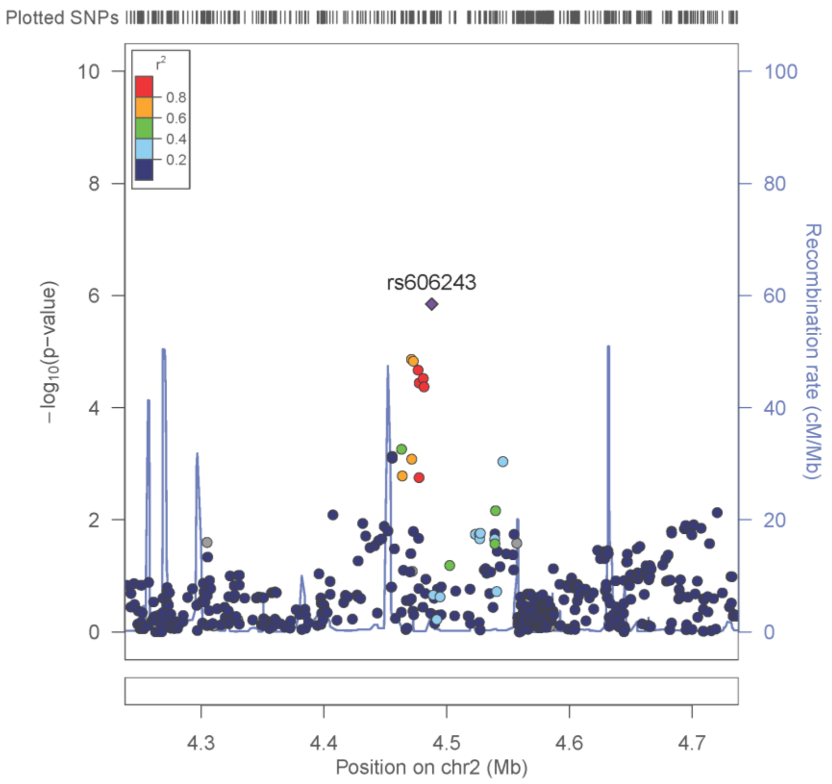

Supplemental Figure 8. Regional association plot of rs17496593. Results from 500 kb regional association plot for fasting glucose change over time centered, at rs17496593. The x axis denotes genomic position and the y axis denotes the –log(P-value) and recombination rate (blue line). The purple diamond symbol represents the most-associated SNP rs17496593 within the region. The color of each symbol indicates its LD value with the top SNP based on the HapMap2 CEU sample.

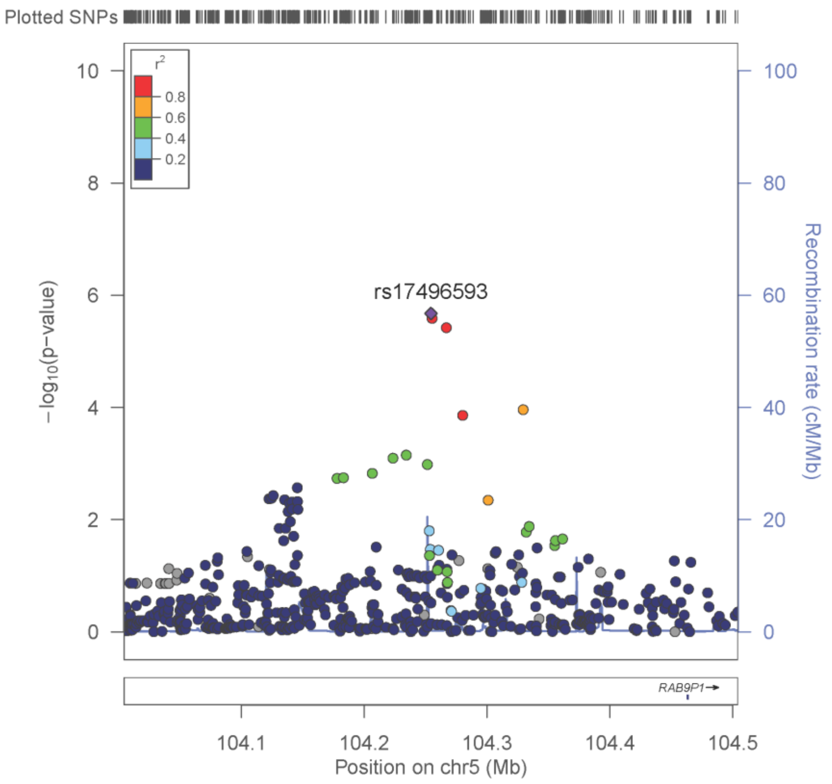

Supplemental Text.

BHS. The Busselton Health Study was approved by the Sir Charles Gairdner Hospital Human Research Ethics Committee and the University of Western Australia Human Research Ethics Committee. There was no requirement for review of this reanalysis.

The **Colaus Study** was reviewed and approved by the Institutional Review Board. The institutional Ethics Committee of the University of Lausanne, which afterwards became the Ethics Commission of Canton Vaud (www.cer-vd.ch) approved the baseline CoLaus study (reference 16/03, decisions of 13th January and 10th February 2003). The approval was renewed for the first (reference 33/09, decision of 23rd February 2009), the second (reference 26/14, decision of 11th March 2014) and the third (reference PB_2018-00040, decision of 20th March 2018) follow-ups. The full decisions of the CER-VD can be obtained from the authors upon request. The study was performed in agreement with the Helsinki declaration and its former amendments, and in accordance with the applicable Swiss legislation (LRH 810.30, approved by the Swiss Federal Parliament on 30th of September 2011). All participants gave their signed informed consent before entering the study. This protocol was not reviewed specifically as this analysis is covered by the consent that the participants give at each follow-up

DESIR. The DESIR was approved by 1. the ethics committee of the Bicetre Hospital, Kremlin Bicetre, France 2. CNIL (Commission Nationale d'Informatique et Liberté), a French commission for data protection. 3. from INSERM who was the 'promotor' of the study. There was no requirement for review of this reanalysis.

ERGO. The Rotterdam Study has been approved by the Medical Ethics Committee of the Erasmus MC (registration number MEC 02.1015) and by the Dutch Ministry of Health, Welfare and Sport (Population Screening Act WBO, license number 1071272-159521-PG). The Rotterdam Study has been entered into the Netherlands National Trial Register (NTR; www.trialregister.nl) and into the WHO International Clinical Trials Registry Platform (ICTRP; www.who.int/ictrp/network/primary/en/) under shared catalogue number NTR6831. All participants provided written informed consent to participate in the study and to have their information obtained from treating physicians. There was no requirement for review of this reanalysis.

The **Framingham heart study** was reviewed and approved by the Boston University Medical Campus Institutional Review Board (IRB) and there was no requirement for review of this reanalysis.

The **HBCS** was approved by the Ethical Committee at Helsinki University Hospital and there was no requirement for review of this reanalysis.

KORA. KORA was approved by the "Ethik-Kommission der Bayerischen Landesärtzekammer". And it was not reviewed specifically for this study, however the consent covers this study. The approval Number is 06068.

PREVEND. The original PREVEND study was approved by the Medical Ethnics Review Board, the University Medical Center (METc UMCG). https://metcgroningen.nl/compositionmetcumcg/ provides the details. This protocol was not reviewed specifically as there was no requirement for review of this reanalysis.

SARDINIA. All participants gave informed consent, with protocols approved by the institutional review boards of the University of Cagliari, the National Institute on Aging, and the University of Michigan. There was no requirement for review of this reanalysis.
